# Congenital malaria: rare but potentially fatal

**DOI:** 10.2217/17455111.2.2.235

**Published:** 2008-03-28

**Authors:** Whitney E Harrington, Patrick E Duffy

**Affiliations:** 1 Seattle Biomedical Research Institute, Malaria Program, Department of Pathobiology, University of Washington, Seattle, WA, USA. Tel.: +1 206 256 7450; Fax: +1 206 256 7229; whitney.harrington@sbri.org; 2 Tel.: +1 206 256 7311; Fax: +1 206 256 7229; patrick.duffy@sbri.org

**Keywords:** artesunate, chloroquine, congenital malaria, *Plasmodium falciparum*, *Plasmodium vivax*, pregnancy malaria

## Abstract

Congenital malaria is rare and usually indolent but can be fatal. Mortality risk is high in newborns with *Plasmodium falciparum* born to nonimmune women, who typically present at birth or soon thereafter. Semi-immune women are less likely to transmit malaria, and their children often become ill weeks after delivery with less severe symptoms. Cases in the USA usually trace to semi-immune immigrant mothers whose last exposure to malaria may have preceded the pregnancy, leading to misdiagnoses. Congenital malaria may be under-recognized in malaria-endemic areas since parasitemia occurring after the first week of life is usually attributed to mosquito transmission. Malaria prophylaxis and the absence of fever during pregnancy do not preclude congenital malaria in a newborn. Quinine plus clindamycin is commonly used to treat *P. falciparum* congenital malaria, and chloroquine is used to treat other malaria parasites, such as *Plasmodium vivax*. Severe cases should be managed with intravenous quinine (available as its enantiomer quinidine in the USA) or with intravenous artesunate, which was recently approved for investigational use by the US FDA. Primaquine is not required for infants with congenital *P. vivax* or *Plasmodium ovale*, but should be offered to their mothers after excluding G6PD deficiency.

**Figure 1.**
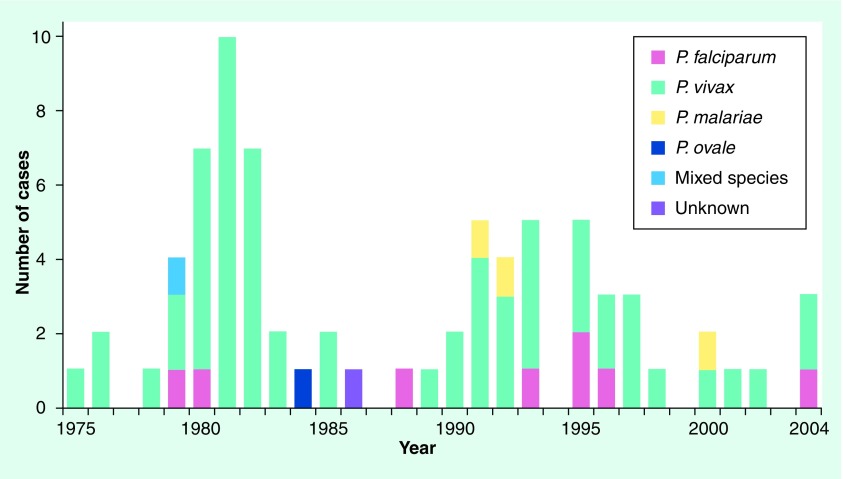
Annual number of congenital malaria cases reported in the USA from 1975 to 2004.

**Figure 2.**
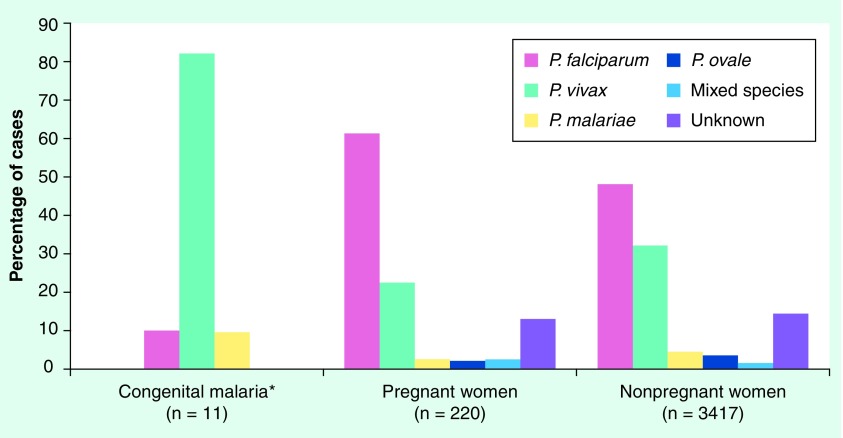
Malaria cases in US infants and women stratified by parasite species, 1997–2004. *No reported cases of congenital malaria were due to *Plasmodium ovale*, mixed species or unknown species.

**Figure 3.**
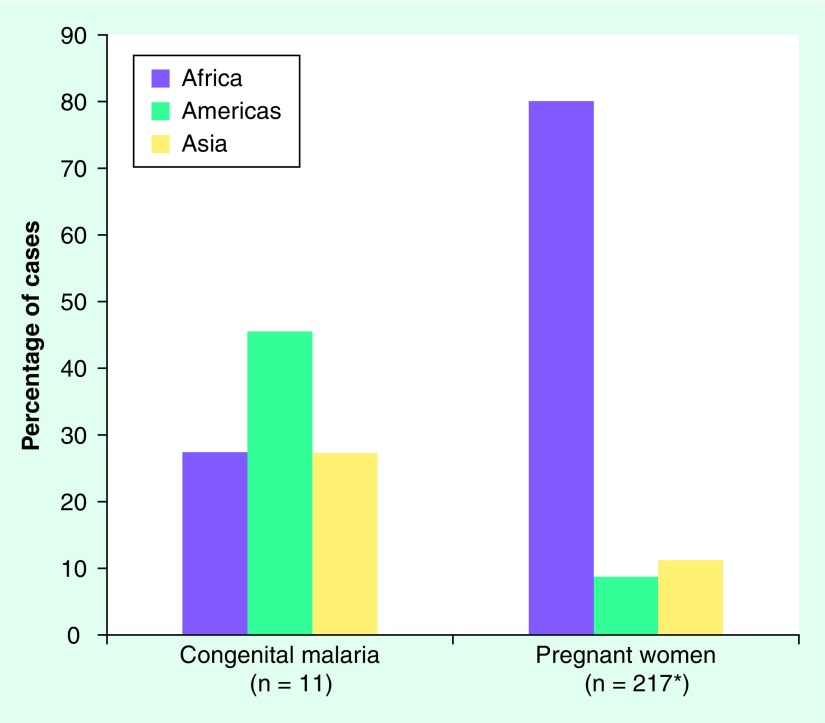
Malaria cases in US infants and pregnant women stratified by the geographic origin of the mother, 1997–2004. *Three cases where origin was unknown were excluded.

Malaria will cause half a billion clinical episodes and one million deaths this year, with most deaths occurring in young African children. In areas of stable transmission, malaria has a distinct epidemiologic signature at the mother–newborn interface: pregnant women are relatively susceptible and newborns are relatively resistant [1]. Congenital malaria, defined as parasitemia transmitted from the mother to the child *in utero* or peripartum, is rare. Since the deadliest malaria parasite, *Plasmodium falciparum*, sequesters in the maternal vascular spaces of the placenta, sometimes at extraordinarily high densities, the infrequent occurrence of congenital malaria cases has puzzled observers for more than a century. Despite the general observation that congenital malaria is uncommon and often indolent in its appearance, its true incidence is controversial and its outcome may be rapidly fatal when the mother lacks immunity. Clinicians should be alert to the possibility of congenital malaria in febrile neonates when the mother is from or has recently traveled to an endemic area, even if she reports no illness during pregnancy (Box 1).

## Pregnancy malaria & congenital transmission

Pregnancy increases susceptibility to *P. falciparum* and most [2–4] but not all studies [5] suggest that it increases susceptibility to *Plasmodium vivax* as well. According to the few available studies, susceptibility to the less common malaria species *Plasmodium malariae* and *Plasmodium ovale* remains unchanged during pregnancy [6,7]. All four human malaria parasites can cause congenital malaria.

*P. falciparum* assumes a novel phenotype during pregnancy that may explain maternal susceptibility. Mature parasite stages sequester in the intervillous (maternal) vascular spaces of the placenta by binding to the receptor chondroitin sulfate A (CSA) [8]. CSA-binding parasites are common in pregnant women but uncommon in nonpregnant individuals. Women lack immunity to the CSA-binding parasite before their first pregnancy, which might explain why primigravidae are highly susceptible [9]. Women who are exposed to malaria become resistant over successive pregnancies as they acquire antibodies against the placental CSA-binding parasites [9]. In areas of stable malaria transmission, multigravidae typically control parasitemia before symptoms or sequelae develop. Younger age and HIV infection also increase susceptibility to pregnancy malaria [10].

The gravidity-specific pattern of susceptibility is not observed among nonimmune pregnant women. These women are at high risk of severe syndromes and death if they become infected, regardless of gravidity, and abortion or fetal death can occur during acute febrile or severe malaria episodes [11,12]. Among semi-immune women in areas of stable transmission, severe maternal anemia and low birth weight are common sequelae of infection during pregnancy [13], and these contribute to significant maternal and infant mortality. Placental malaria may also modify parasitemia risk in the offspring in a gravidity-dependent manner: in Tanzania, placental malaria was recently reported to decrease parasitemia risk in offspring of first-time mothers but to increase risk in offspring of multigravidae, and these effects persisted throughout infancy [14]. To prevent these poor outcomes, the WHO recommends that pregnant women throughout Africa receive intermittent presumptive treatment (IPT) and use insecticide-treated bed nets to reduce the risk of placental malaria, which might also reduce the risk of congenital malaria.

Similar to *P. falciparum*, *P. vivax* has been reported to be more common during first rather than later pregnancies [3,4], although the basis for this epidemiology is not known. Mature stages of *P. vivax* do not sequester in the placenta or other vascular beds, nor do *P. malariae* or *P. ovale* have this property. Mature stages of *P. vivax, P. malariae* and *P. ovale* circulate freely in peripheral blood, and are therefore frequently detected in peripheral blood smears, unlike *P. falciparum* in which only the young parasite stages appear in peripheral blood smears.

Congenital malaria is caused by blood-stage malaria parasites that are transmitted from mother to child. Pre-erythrocytic stages of the parasite – including the sporozoite form inoculated by the mosquito, and the liver stage forms that develop in hepatocytes during the first week of infection – are not known to cross the placenta nor to cause congenital malaria. Therefore, treatment of congenital malaria is intended to eradicate blood-stage and not liver-stage parasites in the newborn.

The mother’s history can be misleading for clinicians who are assessing an infant with congenital malaria, because malaria parasites can persist in asymptomatic individuals. According to a review of all US cases between 1966 and 2005, many mothers who deliver a child with congenital malaria report no exposure and no symptoms during the pregnancy [15]. A large proportion of congenital malaria cases in the US occur among immigrant women who did not travel to an endemic area during their pregnancy [16–29]. *P. falciparum* can persist for months to years [30] and *P. malariae* can persist for decades as blood-stage infections (the stage that multiplies in red blood cells). *P. vivax* and *P. ovale* form hypnozoites in the liver that can relapse after months or sometimes years to cause new blood-stage infections.

Many US mothers who transmit malaria to their children deny having fevers or other illnesses during pregnancy [15], suggesting that congenital malaria may arise even when women have low-density parasitemia (i.e., parasitemia at a level too low to cause symptoms). Similarly, congenital malaria can occur in offspring of mothers who report adherence to malaria prophylaxis during travel or during residence in endemic areas [12,31,32]. Clinicians should be alert to the possibility that congenital malaria can arise even when the mother reports neither malaria episodes nor other febrile illnesses during pregnancy (Table 1).

The mode of parasite transmission from mother to newborn is usually uncertain. Older literature documents abortuses and stillbirths with parasites or parasite pigment in the spleen or liver [11]. In such cases, the parasite was likely to have been transmitted *in utero*. Transmission might also occur across the placental barrier during labor, or as a result of newborn contact with infected maternal blood during parturition.

Maternal blood is sometimes transferred to the fetal circulation during healthy pregnancy, but this is less frequent than transfers of blood from the fetus to the mother. By typing blood group antigens (ABO or Rh) in discordant mother–newborn pairs, fetal red cells have been detected in 41.5% of maternal samples [33], while maternal cells have been variously detected in 10.5% of cord blood samples [34] and 3.6% [33] or 43% of neonatal blood samples [35]. The majority of maternal cells may be cleared from the fetal circulation within the first few days of life – in one series, only two neonates (1.9%) remained with detectable maternal red cells in their blood at day 3 compared with 10.5% at delivery [34].

Placental malaria may or may not disrupt the placental barrier and alter the rate of mother-to-child transmission. One study reported that cord-blood parasitemia was associated with features of chronic placental malaria such as intervillous inflammation [36]. However, placental transfer of micronutrients such as folacin and cobalamin is unchanged during malaria [37]. Fetal levels of maternal molecules that do not normally cross the placenta may (placental alkaline phosphatase) [38] or may not (ergothionine) [39] increase during placental malaria. Thus, it remains unclear whether placental malaria disrupts the placental barrier.

## Worldwide prevalence of congenital malaria

Reports on congenital malaria have suggested a wide variation in prevalence among populations residing in areas of stable transmission (Table 2) [12,31,32,38,40–61]. For example, a single multicountry study from Africa reported prevalence values ranging from 0 to 23% depending on the site [45]. These differences were not related to the malaria transmission intensity in the general population, nor to antimalarial prophylaxis usage [45].

However, large studies conducted at established research centers have uniformly found that congenital malaria defined by Giemsa-stained blood smear is rare in areas of high malaria transmission. Garnham, who authored the standard illustrated guide to malaria parasites, did not observe parasitemia during the first week of life in any of 146 neonates born to Kenyan women infected with *P. falciparum* at delivery [47]. Bruce-Chwatt observed no peripheral parasitemias in 567 Nigerian newborns on the first day of life, and only one parasitemia during the first week of life; by contrast, 27.4% of their mothers were parasitemic at delivery [43]. Similarly, McGregor observed no parasitemias within the first day of life in 147 Gambian newborns of mothers with infected placentas [60]. Covell, who reviewed the world literature in 1950, estimated that congenital malaria occurs in 0.3% of children born to infected women with high levels of immunity [62].

Among offspring of semi-immune women who deliver in nonendemic areas, congenital malaria typically appears several weeks after birth. Presumably, offspring in malaria endemic areas would similarly manifest congenital malaria several weeks after birth, but in these cases, congenital malaria cannot easily be differentiated from mosquito-borne infection. For this reason, researchers in endemic areas have attempted to use more sensitive approaches than Giemsa-stained blood smear to detect parasites in cord or newborn blood, such as PCR detection of parasite DNA or RNA [51].

Researchers using PCR assays report detection of parasites in a considerable proportion of cord blood samples, ranging from 10.6% [38] and 32.0% [59] of cord blood samples in coastal Kenya to 19.8% in Malawi [50] and 45.9% of cord blood samples donated by infected women in Gabon [51]. Since PCR is highly sensitive, contamination of cord blood by minute amounts of placental blood could cause false-positive PCR results. Furthermore, PCR-based assays are simply an amplification of nucleic acids, and it is possible that free nucleic acids are circulating independently of viable parasites [63]. For these reasons, prevalence studies using PCR assessment of cord blood may overestimate the true rate of congenital malaria in endemic settings. Notably, PCR only detected parasites in one of 298 peripheral blood samples collected from Tanzanian neonates within the first week of life [40], a much lower prevalence compared with the PCR studies of cord blood.

Genetic discordance has supported the idea that parasites detected by PCR in cord blood do not reflect contamination of samples by placental blood. Discordance in parasite species [59], parasite density [38] and parasite genotypes [38,51], suggest that maternal and fetal parasites may be distinct. These findings further imply that many cases of mother-to-child transmission are occurring *in utero* and not at the time of delivery, since transmission at the time of delivery would be expected to yield genetically identical parasites in placental and cord blood samples. Nevertheless, the relevance of cord blood parasites detected by PCR to symptomatic congenital malaria or neonatal outcomes is unclear. Just as maternal erythrocytes are cleared from the neonatal circulation during the first days of life, so too have parasites been observed to clear from neonatal blood in endemic areas [54].

Congenital malaria in nonimmune populations has been studied less frequently, but the available data consistently suggest that mother-to-child transmission is more likely in such cases, compared with semi-immune populations. On the Thai–Burmese border, where malaria transmission is low and women therefore have low levels of immunity, three congenital malaria episodes were documented among 213 deliveries [53]. This cohort included women who attended antenatal clinics that offered weekly malaria screening and treatment for all positive cases, which likely decreased the incidence of congenital infection. In colonial Nigeria, three clinical congenital malaria cases occurred among a sequential series of 37 offspring born to expatriate women, many of whom reported taking antimalarial prophylaxis during pregnancy [12]. In the early 1900’s, when malaria was endemic in the USA, Thibault reported four cases of congenital malaria (including one infected stillbirth) among 249 newborns delivered by infected women [61]. Although these studies are relatively small, the incidence of congenital malaria, ranging from 1.4 to 8.1%, is fairly consistent across these diverse populations.

## Incidence of congenital malaria in the USA

Similar to observations elsewhere, the reported incidence of pregnancy malaria in the USA far exceeds the incidence of congenital malaria. Congenital malaria in the US is a rare phenomenon (Figure 1), with only 81 cases reported between 1950 and 2004 [15–17,21–29,64–80]. Between 1997 and 2004, 11 congenital malaria cases, 220 pregnancy malaria cases and 3417 malaria cases in nonpregnant women were reported in the USA (Figure 2). These values only include those reported through the CDC surveillance system, a passive system that relies on voluntary reporting of laboratory-confirmed malaria cases.

In recent years (1966–2005), congenital malaria in the USA has primarily affected immigrant families. In many of these cases, the last exposure to malaria occurred before pregnancy [15]. For cases reported between 1991 and 2004, 44% of mothers reported that they did not travel to an endemic area during their pregnancy. Clinicians need to be alert to the fact that many mothers of children with congenital malaria were not exposed to malaria during pregnancy.

The mother’s time-to-last-exposure also reflects the persistence of malaria parasites in the human host. Between 1966 and 2005, 85% of mothers were exposed within 1 year prior to delivery [15]. However, the range of time between last reported exposure and delivery varied widely – from 36 h to 12 years – emphasizing that even a remote history of travel to an endemic area should be considered a risk factor [15,64]. The woman reporting a last exposure 12 years before delivery was infected with *P. malariae*, which can persist for decades in asymptomatic individuals [16]. One woman whose child developed *P. falciparum* congenital malaria reported her last exposure in Haiti 7 years before delivery, which would be an unusually long period for this parasite species to persist, if true [15,67].

Since *P. falciparum*, but not other species concentrate in placental blood, one might expect that falciparum malaria would disproportionately cause congenital malaria, but this is not the case. In cases reported in the USA between 1997 and 2004, the distribution of parasite species differs substantially between congenital malaria, pregnancy malaria and nonpregnancy malaria cases (Figure 2) [19,20,24–29]. Based on CDC surveillance data, pregnant women are significantly more likely to present with *P. falciparum* infection compared with infants with congenital malaria (Chi square, p = 0.0007) or to nonpregnant women (p = 0.0001). Conversely, *P. vivax* disproportionately appears as a cause of congenital malaria in US cases reported to the CDC (82%), followed by *P. falciparum* (9%) and *P. malariae* (9%). Only one case of *P. ovale* congenital malaria has been reported since 1966 [70]. These figures are similar to those reported from a survey of 27 cases in Thailand, where 81.5% of congenital malaria cases were due to *P. vivax* and 18.5% were due to *P. falciparum* [81].

This may have any of several hypothetical explanations, including:

• *P. vivax* has greater transmission potential, for example since it causes less placental inflammation;
• Women with *P. falciparum* have greater systemic immunity, which is transplacentally transferred and protects the fetus;
• Women with *P. vivax* are less likely to receive treatment that eradicates infection, due to persistent liver stages or to submicroscopic parasitemia;
• *P. falciparum* is more likely to become symptomatic and, therefore, prompt treatment during pregnancy [82];
• Antimalarial drugs such as quinine and mefloquine that may be used to treat malaria during pregnancy can cross the placenta and may contribute to clearing parasites from fetal blood [83,84].

The geographic origins of cases in the USA generally parallel immigration patterns. An increase in congenital malaria cases starting in 1979 reflected the influx of immigrants and refugees from South-East Asia [64,85], and subsequent increases from Latin America [64]. Currently (1991–2004), the largest number of US cases originate from Central America, followed by South Asia. Interestingly, the geographic origins of malaria cases in pregnant women are also significantly different from those of congenital cases (Chi square, p = 0.0002). Pregnant women were more likely to have acquired their infection in Africa, whereas congenital malaria cases originated from non-African sources (Figure 3). This may be due to differences in immune status of the mothers, genetic factors, transmissibility of different parasite species or reporting bias.

## Clinical presentation & mortality

The severity of disease and the age of onset in the newborn are highly dependent on maternal exposure history and, therefore, immunity. Unlike congenital malaria cases reported in US immigrant families [15], offspring of nonimmune women are at significant risk of severe disease and death [12,53,61]. This suggests that passive transfer of maternal antibodies to the fetus can confer a strong degree of protection, but this has not been proven.

### Congenital malaria in offspring of nonimmune women 

Infants of nonimmune women develop symptoms at a younger age, and are at significant risk of serious morbidity and mortality from congenital malaria, particularly when infected by *P. falciparum* (Box 2). Among 175 pregnancies complicated by malaria at a Karen–Burmese refugee camp in Thailand, congenital malaria occurred in three newborns, including two who were symptomatic at birth: one newborn with *P. falciparum* died of severe malaria within 6 days of life [53]. Cases in nonimmune populations usually present at birth or within the first week of life [12,86]. In a series of 37 expatriates delivering in colonial Nigeria, three offspring presented with congenital malaria shortly after birth, with symptoms that included fever, vomiting, pallor, convulsions, pulmonary edema, hepatosplenomegaly and jaundice [12]. All three of these newborns died within 1 week of birth. Among ten congenital malaria cases occurring in nonimmune offspring from these and other series, eight were infected with *P. falciparum* and two with *P. vivax,* and the average time to first symptom was 1.3 days of life [11,12,53,61,86]. Of these ten newborns, six died, and all the deaths occurred among those infected with *P. falciparum.* In the series by Wickramasuriya from Sri Lanka, five stillbirths were also documented to have been infected *in utero* [11].

### Congenital malaria in offspring of semi-immune immigrants

In the USA, congenital malaria cases have been limited to immigrant families over recent years. These women acquired their infection before immigration or during subsequent travel, but in most cases experienced few or no symptoms during pregnancy, suggesting that they had immunity sufficient to suppress clinical signs of malaria (Boxes 3 & 4).

According to a recent review of cases between 1966 and 2005 [15], symptoms first arose at a median age of 21.5 days, but this varied significantly based on parasite species. Symptoms arose at 53 days of age on average for infants with *P. malariae*, and at 25 days of age for infants with *P. vivax* or *P. falciparum* [15]. Between 1991 and 2004, the average age at first symptom was 26 days, and at diagnosis was 32 days. The longest period between first symptom and diagnosis was 56 days.

Among US cases occurring between 1991 and 2004, the most common finding was fever, followed by pallor or anemia, thrombocytopenia and poor oral intake (Table 3). Other symptoms included cough, splenomegaly, hyperbilirubinemia, irritability, hepatomegaly, vomiting and lethargy. Hepatomegaly, splenomegaly and irritability were more commonly reported to the CDC as positive findings before 1991 [64], and the difference between the two reporting periods could be related to changes in the geographic origin of cases or to reporting bias. Misdiagnosis is frequent. Anemia is often misattributed to Rh or ABO incompatibility [87], and the triad of fever, anemia and splenomegaly to one of the TORCH syndromes (toxoplasmosis, other [syphilis], rubella, cytomegalovirus and herpes simplex) or to neonatal sepsis [64,88]. Presumed sepsis was the most common diagnosis at first evaluation, emphasizing the large overlap in presenting symptoms. One case of nephrotic syndrome due to congenital *P. malariae* has been described in a baby born to a heroin addict who had parenterally acquired malaria [89].

Preterm newborns with congenital malaria may have an altered presentation. Symptoms may appear earlier, either at birth or within the first week of life. The earlier presentation has been speculatively attributed to a diminished level of passively transferred antibody [90,91]. Fever is less common than in term infants, and apnea, bradycardia and thrombocytopenia can occur [90,91].

### Congenital malaria in areas of malaria transmission

Offspring of semi-immune women residing in endemic settings are usually protected from clinically significant malaria for the first 2 to 3 months of life [92]. As noted previously, the prevalence of mother-to-child transmission is unclear in endemic areas, but patent parasitemia and symptomatic malaria are rare in newborns of semi-immune women [43,47,48,56,60]. Passively transferred protective maternal antibodies are presumed to contribute, at least in part, to this window of malaria resistance [92]. After the first week of life, congenital malaria is difficult to distinguish from mosquito-transmitted malaria in these settings, and therefore the true incidence of congenital malaria remains a focus for future research. Although rare, clinical congenital malaria in offspring of semi-immune women may present with symptoms similar to those in other congenital malaria cases [49].

## Diagnosis

Congenital malaria should be entertained as a diagnosis in an infant with suggestive symptoms including fever or thrombocytopenia [93], and whose mother has a history of recent or distant malaria exposure. Clinical suspicion can then be confirmed through laboratory studies. Laboratory detection of congenital malaria relies upon Giemsa-stained blood smear. Both thick and thin blood smears are used and the presence of parasites within red blood cells is confirmed through visual inspection by a trained microscopist.

More than one blood smear may be needed to diagnose congenital malaria, as parasite density tends to be low and the chance of failing to detect parasites on a single smear is high [91,94]. In addition to examining peripheral blood of the infant for parasites, many studies have also examined cord blood for parasites. This eliminates the requirement for blood draw from the baby. However, contamination with infected maternal blood is a concern [48], and possibly as a result positive cord blood smear often does not lead to peripheral parasitemia or clinical malaria in the newborn.

In addition to blood smear, highly sensitive and specific PCR-based assays have been developed to make species-specific determinations [95,96], but these assays are not widely available. Rapid diagnostic tests (RDTs) for malaria use immunochromatographic formats, and an RDT was recently approved by the US FDA, but malaria antigens may cross the placental barrier independently of parasites, making specificity an issue [97,98]. The approved RDT detects two parasite proteins: aldolase, which is present in all species of malaria, and HRP2, which is unique to *P. falciparum* [99]. Of note, preliminary studies have suggested that some RDTs may not be useful for diagnosis in newborns [100].

Separately, because HIV increases the risk of malaria during pregnancy [101], congenital malaria diagnosis should prompt additional assessment for HIV in the mother and preventive measures as indicated in the infant.

## Treatment

Unfortunately, very little is known about the pharmacokinetics and pharmacodynamics of antimalarials in neonates. To date, no randomized trials of treatments for congenital malaria have been reported, and therefore treatment guidelines are largely the product of case reports, field experience and extrapolation from older children. Early treatment is essential. In many cases, pediatric preparations may not be available, and thus adult preparations, dissolved in liquid, may need to be substituted.

Treatment regimens are based on parasite species, region where the infection was acquired (parasite resistance profile), and the severity of disease (Table 4) [102,201]. Treatment of symptomatic congenital malaria in the US should be informed by the most recent CDC guidelines for treatment of pediatric malaria [202].

Chloroquine is commonly used for congenital malaria due to *P. vivax* or chloroquine-sensitive strains of *P. falciparum* [202]. Quinine sulfate plus clindamycin is the therapy of choice for uncomplicated chloroquine-resistant *P. falciparum* in the USA [102,202]. Specific dosages should be based upon the weight of the child. Clinicians should be aware of the risks of hypoglycemia during quinine or quinidine therapy, and they should monitor glucose levels. Doxycycline and tetracycline are contraindicated for use in children under 8 years of age, and so should not be used to treat congenital malaria [202].

For severe cases of congenital malaria, such as those complicated by seizures, respiratory distress, hyperparasitemia or severe anemia, therapy should be given parenterally until a fall in parasitemia is noted [201]. Intravenous quinidine gluconate in combination with clindamycin is used in the USA [202], and artesunate was recently approved for investigational use by the US FDA [203].

Intravenous (iv.) or intramuscular (im.) artesunate has been recommended as first-line therapy outside the USA, where alternatives include im. artemether and iv. or im. quinine [201]. Although iv. artesunate has not been studied in neonates, a randomized trial found that it was superior to iv. quinine for reducing mortality due to severe malaria in children (5 vs 11%; p = 0.15) and adults (16 vs 24%; p = 0.0005) [103]. Artesunate is also less likely to cause hypoglycemia than quinine. Sterile abscess is a common complication of im. quinine therapy.

Since congenital malaria is acquired through mother-to-child transfer of blood-stage parasites, treatment with primaquine to eradicate liver-stage *P. vivax* and *P. ovale* is not necessary in the infant [202]. Since 1991, at least four infants in the USA have unnecessarily received primaquine. Mothers of children with *P. vivax* or *P. ovale* congenital malaria should receive treatment with primaquine following delivery, after confirming normal G6PD status. For pregnant women with *P. vivax*, primaquine is contraindicated; weekly chloroquine prophylaxis may suppress parasitemia [104] and should be considered.

Transfusion may be needed to treat severe anemia due to congenital malaria [19,21,29]. Exchange transfusion may be indicated when parasitemias are higher than 10% in *P. falciparum* infections with severe symptoms [202]. In mothers with fever and parasites at delivery, empiric treatment of the newborn for congenital malaria has been recommended [105], but is not universally practiced.

## Conclusion

Congenital malaria is a rare condition. In the USA, congenital malaria is a malady that mainly affects immigrant families. Outcomes are good where passively transferred maternal immunity protects the child, but can be fatal when *P. falciparum* is transmitted by nonimmune women to their offspring. Clinical suspicion of malaria is often low as the mothers’ exposure to malaria may be distant and many mothers do not report fever or illness during pregnancy. Fever is the most common presenting symptom in the infant. Treatment should be tailored to the drug-susceptibility profile of the parasite, according to the species and country of origin. *P. vivax* congenital malaria does not require primaquine treatment of the newborn, as congenital malaria only involves blood-stage parasites.

## Future perspective

Treatment recommendations may change as drug susceptibility profiles of parasites around the world evolve, including the possible spread of chloroquine-resistant *P. vivax* [202]. Clinicians should refer to current CDC pediatric treatment guidelines when managing an infant with malaria.

The frequency, timing and mode of mother-to-child transmission remain poorly defined, and merit further study. Similarly, the long-term consequences of congenital malaria, particularly in endemic areas where the condition is rarely recognized and could modify malaria outcomes in the child, are unknown but are the focus of ongoing research.

**Table 1.** Proportion of US cases of congenital malaria* in which the mother had evidence or not of malaria during pregnancy.

Indicator	Percentage of cases documented
Malaria diagnosis during pregnancy	33
Fever or malaria diagnosis during pregnancy	45
Blood smear positive or symptomatic at delivery	21
Blood smear positive at child’s diagnosis^‡^	18
Malaria not diagnosed before child’s diagnosis	58

*Results of CDC reported congenital malaria cases, 1991–2004 (n = 33). ^‡^One additional mother was positive by PCR but negative by blood smear.

**Table 2.** Frequency of parasites in maternal peripheral, placental, cord and neonatal peripheral blood in studies from around the world.

Author	Year	Location	Immune status	Maternal peripheral	Placental	Cord blood	Neonatal peripheral*	Methodology	Ref.
Africa									
Fischer	1997	sub-Saharan survey	Semi-immune	0.15 (0.04–0.30)			0.07 (0.0-0.23)	BS	[45]
West Africa									
Jones	1950	Lagos, Nigeria	Nonimmune			0.074	0.037	BS	[12]
Bruce-Chwatt	1952	Lagos, Nigeria	Semi-immune	0.274	0.223		0.002	BS	[43]
Ezeoke	1985	Calabar, Nigeria	Semi-immune	0.084	0.262	0.168	0.075	BS	[44]
Akindele	1993	Ibadan, Nigeria	Semi-immune				0.237	BS	[41]
Ibhanesebhor	1995	Benin City, Nigeria	Semi-immune				0.059	BS	[49]
Mukhtar	2005	Lagos, Nigeria	Semi-immune	0.190	0.150	0.144	0.134	BS	[54]
Obiajunwa	2005	Ile-Ife, Nigeria	Semi-immune		0.567	0.542	0.467	BS	[56]
Runsewe-Abiodun	2006	Sagamu, Nigeria	Semi-immune				0.174	BS	[58]
Reinhardt	1978	Abidjan, Ivory Coast	Semi-immune	0.394	0.333	0.217		BS	[57]
McGregor	1984	Banjul & rural villages, The Gambia	Semi-immune	0.317	0.202		0 (n = 147)^‡^	BS	[60]
Garin	1985	Franceville, Gabon	Semi-immune	0.364	0.330	0 (n = 1128)		BS	[46]
Kassberger	2002	Lambarene, Gabon	Semi-immune			0.459^‡^		PCR	[51]
Central Africa									
Larkin	1991	Southern Province, Zambia	Semi-immune	0.630			0.290	BS	[31]
Nyirjesy	1993	Nyakunde, Zaire	Semi-immune	0.210	0.330	0.090	0.070	BS	[55]
Redd	1996	Mangochi district, Malawi	Semi-immune	0.150	0.187	0.067		BS	[32]
Kamwendo	2002	Blantyre, Malawi	Semi-immune			0.198 (0.058)		PCR (BS)	[50]
East Africa									
Garnham	1938	Kisumu, Kenya	Semi-immune				0 (n = 400)	BS	[48]
Garnham	1949	Kisumu, Kenya	Semi-immune				0 (n = 146)^‡^	BS	[47]
Bergstrom	1993	Maputo, Mozambique	Semi-immune	0.163	0.124	0.015		BS	[42]
Adachi	2000	Dar es Salaam, Tanzania	Semi-immune				0.003	PCR	[40]
Tobian	2000	Coast Province, Kenya	Semi-immune	0.480		0.320		PCR	[59]
Malhotra	2006	Kwale District, Kenya	Semi-immune	0.364	0.159	0.106		PCR	[38]
Asia									
Marshall	1983	Maliata province, Solomon Islands	Semi-immune	0.061	0.055	0.028	0.006	BS	[52]
McGready	2004	Northwest border, Thailand	Nonimmune	0.095	0.065	0.011		BS	[53]
USA									
Thibault	1915	Arkansas, United States	Nonimmune				0.016^‡^	BS	[61]

*Includes neonates tested within the first 7 days of life. ^‡^Includes only neonates born to infected women. BS: Giemsa-stained blood smear.

**Table 3.** Proportion of US cases of congenital malaria with specific signs.

Sign	Percentage of cases documented
Fever	96.7 (n = 30)
Anemia	100.0 (n = 12)
Thrombocytopenia	100.0 (n = 7)

Results represent CDC reported CM cases, 1991–2004, and indicate only cases in which the sign was documented.

**Table 4.** Recommended therapy for children with congenital malaria.

Clinical diagnosis/species	Region infection acquired	Recommended therapy (alternatives not to be administered simultaneously)
Uncomplicated malaria		
*Plasmodium falciparum*	Chloroquine sensitive	Chloroquine phosphate (10 mg base/kg p.o. immediately, followed by 5 mg base/kg p.o. at 6, 24, and 48 h; total dose: 25 mg base/kg)
Hydroxychloroquine (10 mg base/kg p.o. immediately, followed by 5 mg base/kg p.o. at 6, 24 and 48 h; total dose: 25 mg base/kg)		
Chloroquine resistant	Quinine sulfate* (8.3 mg base/kg [=10 mg salt/kg] p.o. t.i.d. × 3 to 7 days) plus clindamycin (20 mg base/kg/day p.o. divided t.i.d. × 7 days)	
Atovaquone–proguanil^‡^ (5–8 kg: 2 peds tabs p.o. q.d. × 3 days; 9–10 kg: 3 peds tabs p.o. q.d. × 3 days; 11–20kg: 1 adult tab p.o. q.d. × 3 days (adult tab = 250 mg atovaquone/100 mg proguanil, peds tab = 62.5 mg atovaquone/25 mg proguanil)		
Mefloquine^§^ (13.7 mg base/kg [=15 mg salt/kg] p.o. as initial dose, followed by 9.1 mg base/kg [=10 mg salt/kg] p.o. administered 6–12 h after initial dose; total dose = 25 mg salt/kg)		
*Outside the USA* Artesunate (2 mg/kg/daily) plus clindamycin (5 mg/kg/t.i.d.) × 7 days – Shoklo recommendation		
Artemisinin combination therapy – WHO pediatric recommendation		
*Plasmodium malariae*, *ovale* or *vivax*	Chloroquine susceptible	Chloroquine phosphate (see dosage above)
Hydroxychloroquine (see dosage above)		
Severe malaria		
All regions	Quinidine gluconate*^¶^ (iv.; loading dose: 24 mg/kg iv. in 250 ml NS infused over 4 h; maintenance dose: 12 mg/kg iv. infused over 4 h every 8 h for 7 days or until oral therapy started) plus clindamycin (10 mg base/kg loading dose iv. followed by 5 mg base/kg iv. every 8 h. Switch to oral meds when tolerated. For iv. use, avoid rapid administration. Treatment course = 7 days)	
Quinine sulfate (iv.)*^¶^		
iv. artesunate (investigational; contact CDC)		
Outside the USA iv. (or im.) artesunate^¶^ (2.4mg/kg at 0, 12 and 24 h, then 2.4 mg/kg daily for 7 days iv., im. or oral)		
Im. artemether^¶^ (3.2 mg/kg at 0 h, 1.6 mg/kg at 24 h and every 24 h until oral meds tolerated)		
iv. (or im.) quinine*^¶^ (20 mg/kg loading dose given over 4 h, followed at 8 h by 10mg/kg over 2 h, repeated every 8 h; total daily dose 30 mg/kg; switch to oral meds when tolerated)		

Consider exchange transfusion if parasitemia is over 10% or if the patient has altered mental status, nonvolume overload pulmonary edema or renal complications. *Neonates are particularly vulnerable to hypoglycemia when treated with quinine or quinidine. If the mother is also treated with quinine or quinidine, the effect may be compounded. ^‡^The safety and efficacy of malarone (atovaquone–proguanil) for the treatment of malaria in pediatric patients who weigh less than 11 kg has not been established. ^§^The safety and efficacy of lariam (mefloquine) for the treatment of malaria in pediatric patients younger than 6 months of age has not been established. Experience with lariam in infants less than 3 months old or weighing less than 5 kg is limited. ^¶^All intravenous therapy should be switched to oral when tolerated. im.: Intramuscular; iv.: Intravenous; NS: Normal saline; peds tabs: Pediatric tablets; p.o.: Per os; q.d.: Once daily; tab: Tablet; t.i.d.: Three-times per day. Adapted from [106,201,202]

Box 1. **Defining congenital malaria.**

Congenital malaria refers to the transmission of parasites from the mother to the fetus, either *in utero* or during the peripartum period. Parasites transmitted from mother to child may be detected in the cord blood at birth, or in the infant’s peripheral blood. In areas without mosquito transmission, malaria arising in an infant is presumed to be congenital malaria. In endemic countries, parasites must be detected within the first week of life to confidently diagnose congenital infection, as the incubation period of mosquito-transmitted infection is approximately 1 week. Clinical diagnosis of congenital malaria has relied on Giemsa-stained blood smear analysis. Detection of cord-blood parasitemia may be confounded by contamination of the sample with infected maternal blood. Soluble malaria antigen and genetic material can be transferred transplacentally, which may confound antigen- and DNA-detection assays.

Box 2. **Congenital malaria in a refugee camp in Thailand.**

A pregnant Karen woman was diagnosed with *Plasmodium falciparum* malaria. She delivered a boy while still undergoing treatment. At the time of delivery, *P. falciparum* parasites were detected by Giemsa-stained smear in placental blood and in the newborn’s peripheral blood, but not in the cord blood. The infant was symptomatic at birth. Despite treatment, the newborn deteriorated and died of severe malaria on the sixth day of life.

Taken from [54].

Box 3. **Congenital malaria in the USA due to *Plasmodium falciparum.***

In 2004, a baby boy was born in the USA to a woman from Nigeria who was asymptomatic during pregnancy, but who experienced fever during delivery and was diagnosed with *Plasmodium falciparum*. The baby experienced fever shortly after birth that resolved, and blood smears at birth, 24 h and 48 h were all negative. At age 5 and a half weeks the baby presented with 3 days of fever and irritability. Laboratory studies showed anemia, thrombocytopenia and elevated lactate dehydrogenase. Cultures were taken and the baby was started on ceftriaxone for presumed sepsis. A blood film demonstrated 3% *P. falciparum* parasitemia. The baby was treated with oral quinine sulfate and clindamycin and made a full recovery.

Taken from [30].

Box 4. **Congenital malaria in the USA due to *Plasmodium vivax*.**

A 4-week old male infant was hospitalized for fever and decreased oral intake. The infant was the product of spontaneous vaginal delivery and had no malaria exposure history. The infant’s mother had immigrated from Guatemala 1 year prior to the infant’s birth and had experienced a febrile episode that resolved without therapy when she was 6 months pregnant. The infant was was started on ampicillin and gentamicin for presumed sepsis. At admission, the infant was anemic with a hemoglobin of 9.2 g/dl on day 1 and 8.0 g/dl on day 2. Bacterial cultures were negative, and the infant was discharged on day 4. 3 days later, the patient was found to have a hemoglobin level of 6.0 g/dl. Trophozoites and gametocytes of *Plasmodium vivax* (<1% parasitemia) were seen on blood smear. The infant was admitted and received chloroquine therapy and a blood transfusion. The patient recovered fully, and all subsequent blood smears were negative. At the time of malaria diagnosis of the child, the mother had a negative blood smear, but a positive PCR test for *P. vivax*. She was treated with chloroquine and primaquine after being screened for G6PD deficiency.

Taken from [30].

**Executive summary**

• Congenital malaria in the USA primarily affects immigrant families.

• It is frequently misdiagnosed, because symptoms overlap with other infections and the mother often reports no travel and no illness during pregnancy.

• Congenital malaria is more likely when the infected mother has low immunity than when she has high immunity.

• It can be rapidly fatal when a nonimmune woman transmits *Plasmodium falciparum* to her child.

• Congenital malaria tends to appear earlier when the mother lacks immunity and when *P. falciparum* is the causative agent.

• It can occur in offspring of mothers who report adherence to malaria prophylaxis during travel or during residence in endemic areas.

• *Plasmodium vivax* is the most common cause of congenital malaria in the USA, and *P. falciparum* is the most common cause of pregnancy malaria.

• Congenital malaria should be considered as a cause of fever in an infant whose mother has recent or distant travel to an endemic area.

• The diagnosis of congenital malaria is made by Giemsa-stained blood smears, and a single negative blood smear is not sufficient to exclude the diagnosis.

• Treatment should be based on parasite species, region where the infection was acquired (parasite resistance profile) and the severity of disease. Treatment with primaquine to eradicate liver stage *P. vivax* is not indicated for the infant, but should be given to the mother after G6PD testing.
